# Are Individuals With Type 2 Diabetes Metabolically Inflexible? A Systematic Review and Meta‐Analysis

**DOI:** 10.1002/edm2.70044

**Published:** 2025-05-02

**Authors:** Maria Hansen, Kristine Kjær Lange, Martin Bjørn Stausholm, Flemming Dela

**Affiliations:** ^1^ Department of Biomedical Sciences, Faculty of Health and Medical Sciences University of Copenhagen Copenhagen Denmark; ^2^ Department of Physical and Occupational Therapy Copenhagen University Hospital, Bispebjerg and Frederiksberg Copenhagen Denmark; ^3^ Department of Global Public Health and Primary Care University of Bergen Bergen Norway; ^4^ Laboratory of Sports and Nutrition Research Riga Stradins University Riga Latvia

**Keywords:** hyperinsulinemic clamp, metabolic flexibility, type 2 diabetes

## Abstract

**Aim:**

Type 2 diabetes (T2D) is characterised by insulin resistance and possibly by impaired metabolic flexibility, the latter referring to the body's ability to switch between fuel sources. This review systematically examines metabolic flexibility, measured by changes in the respiratory exchange ratio (ΔRER) during hyperinsulinaemic clamps, across lean, overweight/obese, and T2D populations.

**Methods:**

A comprehensive search of PubMed identified 65 studies meeting the inclusion criteria, with 35 using a ~40 mU/m^2^/min insulin infusion rate for accurate comparisons. These studies included 985 participants: 256 lean, 497 overweight/obese, and 232 T2D individuals. The differences in ΔRER between the three groups were meta‐analysed.

**Results:**

Basal RER values did not significantly differ across groups, but insulin‐stimulated ΔRER was higher in lean individuals compared to overweight/obese and T2D groups (ΔRER values 0.10, 0.07 and 0.07, respectively; *p* = 0.037) indicating greater metabolic flexibility in the lean group. However, high statistical heterogeneity in the ΔRER within‐group results (*I* ^2^ values: 92.3%–94.5%) suggests considerable variability among studies. A meta‐regression analysis accounting for age, sex, and BMI indicated that only BMI was significantly associated with ΔRER. Factors contributing to the remaining heterogeneity likely include differences in participant characteristics (e.g., glycaemic control) and study design.

**Conclusions:**

The review highlights the need for standardised data presentation in metabolic studies. Overall, metabolic flexibility appears more influenced by overweight status than T2D per se, challenging the notion of a distinct metabolic inflexibility threshold for T2D.

## Graphical abstract

Lean people are more metabolically flexible compared with overweight patients and those with Type 2 diabetes, but metabolic flexibility is more influenced by weight status than Type 2 diabetes per se. There is a need for standardised data presentation in metabolic studies. No distinct metabolic inflexibility threshold exists for Type 2 Diabetes.

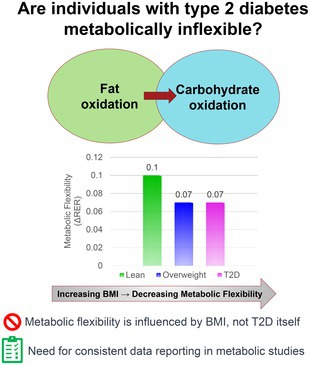

**Summary**

- What is already known?
  ○ Metabolic flexibility is the ability to switch from predominantly fat to carbohydrate oxidation upon insulin stimulation.
- What this study has found?
  ○ Lean people are more metabolically flexible compared with overweight individuals and patients with Type 2 diabetes, but metabolic flexibility is more influenced by weight status than Type 2 diabetes per se.
- What are the implications of the study?
  ○ There is a need for standardised data presentation in metabolic studies, and the use of isoglycaemic clamps is recommended to better reflect the individuals' physiological state. No distinct metabolic inflexibility threshold exists for Type 2 Diabetes.

## 1 Introduction

Type 2 diabetes (T2D) is a complex metabolic disease affecting global health, and its prevalence is increasing worldwide [1]. T2D is the result of the combination of insulin resistance and impaired insulin secretory capacity, and the presence of insulin resistance has been associated with impaired metabolic flexibility, i.e. the capacity to switch between fuel sources. The term metabolic “inflexibility” was introduced in 1999 by Kelley et al., who reported a lesser increase in respiratory quotients (RQ) across a leg (primarily representing skeletal muscle) in response to insulin stimulation in insulin‐resistant (obese) compared to insulin‐sensitive (lean) subjects [2]. This demonstrated less carbohydrate oxidation and therefore relatively higher fat oxidation rates during elevated plasma insulin concentrations. Thus, metabolic flexibility is the ability to switch from predominantly fat to carbohydrate oxidation when changing from an overnight fasted to an insulin‐stimulated or fed state, and the definition of metabolic flexibility has been extended to a systemic level [3]. On the whole‐body, systemic level, metabolic flexibility is usually studied by indirect calorimetry during a hyperinsulinaemic clamp, and the degree of metabolic flexibility is reflected by the change in the respiratory exchange ratio (RER) between the fasted and insulin‐stimulated condition (ΔRER).

Some studies have shown that individuals with T2D have impaired metabolic flexibility compared to lean individuals and compared to body weight and age‐matched overweight individuals without T2D [4, 5, 6]. However, to date, no one has made a comprehensive overview of the data existing on metabolic flexibility in individuals with T2D. Only one systematic review has been conducted on metabolic flexibility [7], but Glaves et al. focused on the association between metabolic flexibility and adipose tissue and included only studies that reported adipose tissue characteristics. Although studies on T2D were included in that review, the disease was not given specific attention. Other narrative reviews on metabolic flexibility have described its relation to insulin resistance [8, 9], more general aspects of metabolic health including response to dietary or exercise interventions [3, 10], and mitochondrial function [11], where metabolic flexibility specifically in T2D only played a minor role.

The term *metabolic inflexibility* is regularly used [8, 9, 12, 13, 14] but no consensus exists on a precise definition; no cut‐off value describes when an individual is metabolically inflexible, which complicates the use of the term *inflexibility*.

In the present review, we collected and compared studies presenting results on metabolic flexibility in individuals with normal weight, overweight/obese, or T2D. We aimed to investigate whether individuals with T2D truly display impaired flexibility to insulin stimulation by a hyperinsulinaemic clamp and if this is associated with the diagnosis of overweight/obesity.

## 2 Methods

### 2.1 Data Sources and Search Strategy

We systematically searched the PubMed database from its inception in 1982 through 21st February 2024 for studies on metabolic flexibility (i.e., insulin‐induced changes in respiratory exchange ratio (ΔRER)) in lean individuals, overweight/obese individuals, and individuals with T2D. The literature search consisted of three separate search strings targeted at the three populations. They consisted of keywords related to (1) lean AND metabolic flexibility AND clamp; (2) overweight AND metabolic flexibility AND clamp; and (3) T2D AND metabolic flexibility AND clamp. The search strings and the PRISMA checklist are provided in Appendix S1. No ethical issues are relevant to this review.

### 2.2 Study Selection Criteria

We included studies reported in the English language that involved at least one of the three human target groups, that is, lean (Lean; BMI < 25 kg/m^2^), overweight (OW; BMI > 25 kg/m^2^) or T2D (as defined in each article) and where indirect calorimetry at basal and during a hyperinsulinaemic euglycaemic or isoglycaemic clamp was performed. We included basal (post‐absorptive) RER data from any study type. In case a clinical trial lacked baseline (before an intervention) results but did provide post‐sham (placebo) RER results, these were included in the analysis. The data had to be presented as ΔRER or be calculable from basal and insulin‐stimulated values. Studies were excluded if the study populations were unclear or mixed (e.g., individuals with impaired glucose tolerance in a T2D group) if a major disease might affect the results (e.g., other types of diabetes), or if the non‐diabetic Lean and OW groups explicitly consisted of first‐degree relations of individuals with T2D. For the sake of comparison, the use of a metabolic chamber, insulin‐infusion rates below 10 mU/m^2^/min, co‐infusions during or major interventions before the hyperinsulinaemic clamp (e.g., an intravenous glucose tolerance test) that might affect the outcome led to exclusion.

### 2.3 Study Selection Process

Two reviewers (MH and KKL) each independently selected the studies. Both reviewers scrutinised the titles/abstracts of all the publications identified in the search and evaluated all potentially eligible articles in a full‐text format. Disagreements were resolved by consensus.

#### 2.3.1 Data‐Extraction and Statistical Analysis

Two reviewers (MH and KKL) extracted participant characteristics: sex, age, weight, body mass index, haemoglobin A1c (HbA1c) and RER.

Both basal and ΔRER were meta‐analysed. Standard errors for meta‐analysis were extracted or estimated from other variance data in a prioritised order by one reviewer (MBS): (1) standard deviation, (2) 95% confidence interval, (3) *p*‐value, (4) median of correlations, or (5) other methods. Another reviewer (MH) carefully checked the work for correctness. The meta‐analyses were conducted with indirect comparisons between the three population groups based on the DerSimonian and Laird random effects model and Mean Difference method [15] in the Stata software program version 18. The potential influence of age and BMI on ΔRER was assessed using the squared Pearson correlation coefficient (*r* ^2^). Additionally, the potential effects of age, sex, and BMI on ΔRER were evaluated through a restricted maximum likelihood meta‐regression analysis with a backward stepwise approach, removing non‐significant variables.

## 3 Results

### 3.1 Study Characteristics

**FIGURE 1:**
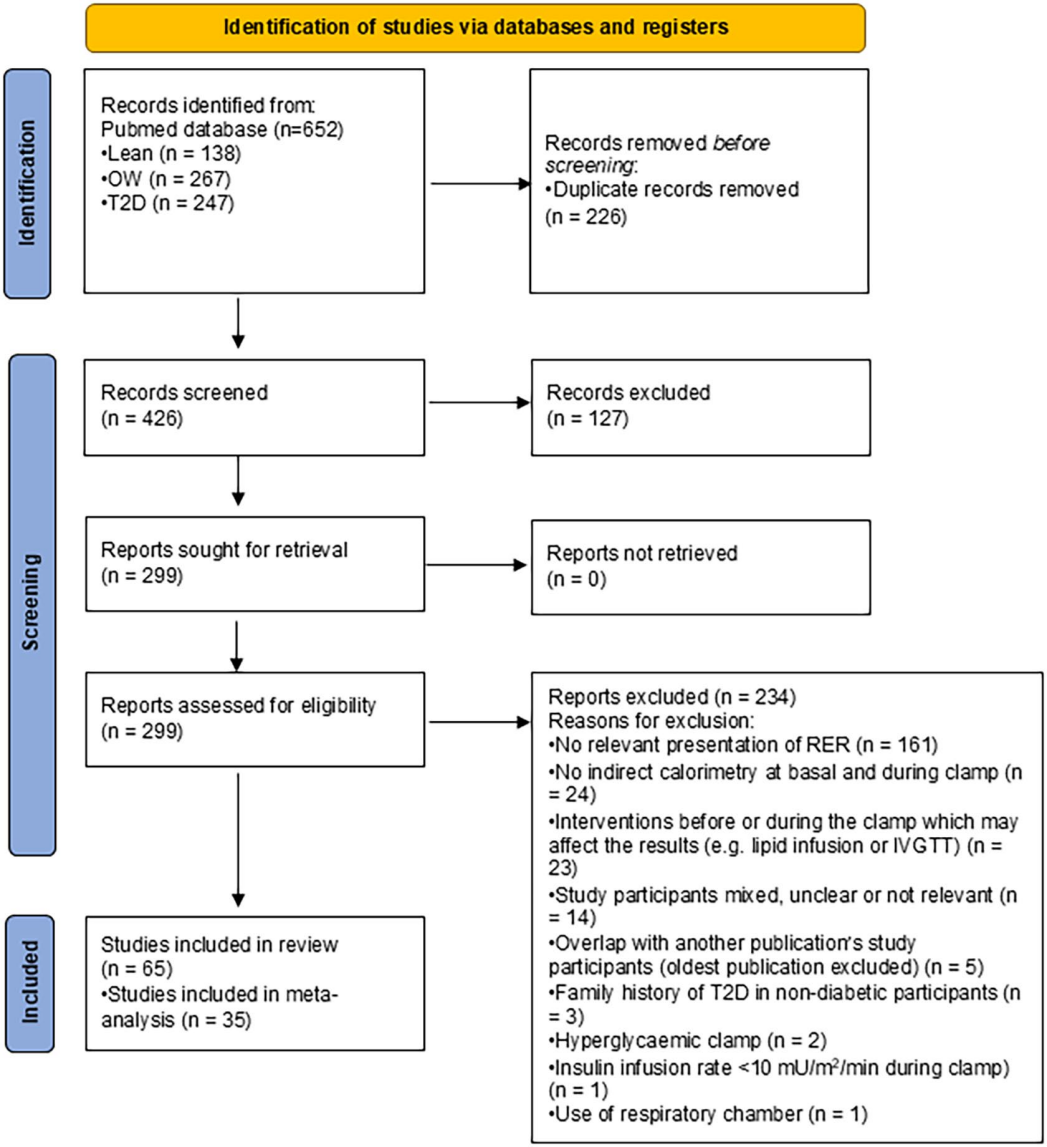
PRISMA flow diagram illustrating the study selection process [16].

Figure 1 shows the PRISMA flow diagram of the identification, screening, and inclusion process [16]. The literature search yielded 652 records, out of which 226 were duplicates. 127 records were excluded by title/abstract, and 299 records were retrieved and assessed in full‐text format. Of the full‐text records, 234 were excluded, mainly due to a lack of relevant presentations of RER (69%). In total, we included 65 studies in this review but chose to focus the analysis on the studies that used an insulin infusion rate of ~40 (i.e., 37–43) mU/m^2^/min for a more accurate comparison (Table 1). The characteristics of studies using < 37 or > 43 mU/m^2^/min are shown in Table S1. Two studies [19, 37] used more than one insulin infusion rate and are included in both tables.

**TABLE 1:** Characteristics of included studies (insulin infusion rate ~40 ± 3 mU/m^2^/min during a hyperinsulinaemic euglycaemic clamp).

Author, year, references	Study type	Participant general characteristics						Clamp			Indirect calorimetry
		Group	Number *n* (m/f)	Age years	Weight kg	BMI kg/m^2^	HbA1c mmol/mol	Insulin infusion rate mU/m^2^/min	Basal RER	ΔRER	Significance *p* value
Adamska, 2013, [17]	Cross‐sectional	Lean	14 (0/14)	26.6	N/A	22.0	N/A	40	0.81	0.06	0.001 ΔRER Lean vs. OW
		OW	16 (0/16)	26.9	N/A	31.2	N/A	40	0.80	0.00	
Adamska, 2014, [18]	Cross‐sectional	Lean	22 (0/22)	24.3	60.7	22.0	N/A	40	0.80	0.08	0.002 ΔRER Lean vs. OW
		OW	26 (0/26)	25.1	85.8	31.0	N/A	40	0.80	0.01	
Bacha, 2021, [19]	Cross‐sectional	Lean	24 (17/7)	16.2	65.2	22.1	37^b^	40	0.82	0.10	N/A
Bergman, 2007, [20]	Clinical trial	Lean	14 (N/A)	28.0	68.2	22.8	N/A	40	0.77	0.09^b^, ^c^	< 0.05 Basal vs. clamp RER
		OW	11 (N/A)	34.6	102.7	35.4	N/A	40	0.76	0.07^b^, ^c^	< 0.05 Basal vs. clamp RER
Brouwers, 2017, [21]	Cross‐sectional	OW	11 (11/0)	57.6	93.8	29.5	N/A	40	N/A	0.13	0.047 ΔRER T2D vs. OW
		T2D	13 (13/0)	63.0	90.4	28.8	N/A	40	N/A	0.09	
Bruls, 2019, [22]	RCT	OW	12 (10/2)	61	N/A	28.9	N/A	40	0.80	0.10	N/A
Bryson, 1996, [23]	Clinical trial	OW	11 (7/4)	47	118.0	40.5	N/A	40	0.83	0.05^b^	N/A
Catalano, 1998, [24]	Cohort	Lean	6 (0/6)	31.8	N/A	20.8	N/A	40	0.84	0.17^b^	N/A
Chomentowski, 2011, [25]	Cross‐sectional	Lean	12 (3/9)	47	65.6	23.7	36^b^	40	0.78	0.15	< 0.01 ΔRER Lean vs. OW and T2D
		OW	17 (6/11)	44	91.5	32.9	37^b^	40	0.80	0.07	
		T2D	11 (5/6)	44	99.6	34.3	60^b^	40	0.80	0.05	
Christ‐Roberts, 2004, [26]	Clinical trial	OW	16 (6/10)	36	N/A	28.0	29^b^	40	0.83	0.09^b^	< 0.001 Basal vs. clamp RER
		T2D	6 (4/2)	45	N/A	28.2	65^b^	40	0.79	0.02^b^	NS Basal vs. clamp RER
de Courten, 2015, [27]	Cross‐sectional	Lean	9 (9/0)	47	N/A	24.5	N/A	40	0.84	0.03	NS ΔRER all groups
		OW	9 (9/0)	44	N/A	29.4	N/A	40	0.88	−0.02	
		T2D	9 (9/0)	46	N/A	30.7	N/A	40	0.86	−0.09	
Croci, 2013, [28]	Cross‐sectional	Lean	15 (10/5)	41	71.2	23.4	N/A	38^b^	0.78	0.11	N/A
Fechner, 2020, [29]^a^	RCT	OW	19 (9/10)	62.2	85.4	29.2	36^b^	40	0.72^b^	0.09	N/A
		OW	21 (10/11)	60.6	86.2	29.5	37^b^	40	0.70^b^	0.10	
Faerch, 2011, [12]	Cross‐sectional	Lean	20 (9/11)	52.4	N/A	23.7	37^b^	40	0.81	0.16	< 0.05 ΔRER between all groups
		OW	31 (22/9)	51.6	N/A	31.5	34^b^	40	0.80	0.12	
		T2D	31 (21/10)	51.5	N/A	32.2	54^b^	40	0.79	0.07	
Heiston, 2022, [30]	Cross‐sectional	OW	47 (8/39)	54.8	105	36.4	N/A	40	0.83	0.02^b^	0.003 Basal vs. clamp RER
Hubinger, 1997, [31]	RCT	T2D	12 (6/6)	53.2	89.1	30.3	89^b^	43^b^	0.77	0.08^b^	N/A
Malin, 2014, [32]	Clinical trial	OW	20 (9/11)	66.4	97.9	34.1	N/A	40	0.83	−0.03	N/A
Mensink, 2007, [33]	Clinical trial	OW	10 (10/0)	57.4	92.7	30.3	N/A	40	N/A	0.08	0.06 ΔRER T2D vs. OW
		T2D	10 (10/0)	61.8	90.2	30.2	56^b^	40	N/A	0.05	
Mingrone, 1997, [34]	RCT	T2D	5 (5/0)	52.2	76.8	26.9	N/A	40	N/A	0.06	N/A
Most, 2016, [35]^a^	RCT	OW^e^	20 (10/10)	38.7	88.3	29.5	33^b^	40	0.78	0.09	N/A
		OW^f^	18 (8/10)	36.1	92.4	29.9	32^b^	40	0.80	0.10	
Okereke, 2004, [36]	Cohort	OW	8 (0/8)	31.6	71.4	26.2	N/A	40	0.87	0.15^b^	N/A
Op den Kamp, 2021, [37]	RCT	T2D	24 (19/5)	64.2	N/A	28.1	51.7	40	0.78	0.09	N/A
Pallubinsky, 2020, [38]	Clinical trial	OW	11 (11/0)	65.7	95.5	30.4	N/A	40	0.80	0.05	N/A
Pedersen, 2017, [39]	RCT	OW	9 (9/0)	24^d^	N/A	30	N/A	40	0.84	0.04^b^	NS Basal vs. clamp RER
Petchey, 2013, [40]	RCT	OW	11 (7/4)	61	N/A	30	40^b^	40	0.79	0.09^b^	N/A
Petersen, 2005, [41]	Cross‐sectional	Lean	7 (N/A)	29	66	21.3	N/A	40^b^	0.79	0.13^b^	N/A
Ravussin, 1983, [42]	Cross‐sectional	Lean	10 (6/4)	38	71.0	N/A	N/A	40	0.80	0.11^b^	< 0.05 Basal vs. clamp RER
		OW	7 (3/4)	31	98.2	N/A	N/A	40	0.82	0.09^b^	< 0.05 Basal vs. clamp RER
Snel, 2012, [43]^a^	RCT	T2D	14 (6/8)	56.1	112.7	37.9	62^b^	40	0.79	0.05^b^	N/A
		T2D	13 (8/5)	53.0	113.5	36.4	62^b^	40	0.84	0.00^b^	
Solomon, 2013, [44]^a^	RCT	OW	10 (4/6)	66	96.9	35.3	41^b^	40	0.86	0.02	N/A
		OW	10 (5/5)	63	97.8	34.8	37^b^	40	0.87	−0.01	
Straczkowski, 2013, [45]	Cross‐sectional	Lean	42 (14/28)	24.2	N/A	22.2	N/A	40	N/A	0.05	0.02 ΔRER OW vs. Lean
		OW	42 (9/33)	26.1	N/A	30.9	N/A	40	N/A	0.02	
Szendroedi, 2014, [46]	Cross‐sectional	OW	10 (5/5)	29	N/A	41.4	34	40	0.79	0.08^b^	NS Basal vs. clamp RER
		T2D	10 (5/5)	59	N/A	35.6	54	40	0.71	0.17^b^	NS Basal vs. clamp RER
Unni, 2009, [47]	Cross‐sectional	Lean	51 (51/0)	21^d^	51.0^d^	18.3^d^	N/A	40	0.85^d^	0.04^b^, ^d^	< 0.001 Basal vs. clamp RER
Veleba, 2015, [48]	RCT	T2D	13 (N/A)	62.0^d^	87.0^d^	30.9^d^	52^d^	43^b^	0.80^d^	0.07^d^	N/A
Vind, 2012, [49]	Cross‐sectional	Lean	10 (4/6)	54.0	N/A	23.2	36^b^	40	0.82	0.17^b^	< 0.05 Clamp RER T2D vs. OW < 0.001 Clamp RER T2D vs. Lean
		OW	10 (6/4)	55.4	N/A	31.1	34^b^	40	0.81	0.15^b^	
		T2D^g^	12 (6/6)	53.5	N/A	29.1	50^b^	40	0.80	0.10^b^	
van de Weijer, 2013, [50]	Cross‐sectional	OW	54 (54/0)	59.8	N/A	29.4	40^b^	40	0.79	0.09	< 0.01 ΔRER T2D vs. OW
		T2D	49 (49/0)	61.2	N/A	29.8	54^b^	40	0.82	0.05	

*Note:* Thirty‐five studies using insulin infusion rates of 37–43 mU/m^2^/min during the clamp in lean and overweight (OW) individuals and patients with type 2 diabetes (T2D) with corresponding RER values. Where possible, we converted insulin infusion rates to mU/m^2^/min (1 μU/mL = 6.00 pmol/L) [51]. Body area was calculated by the formula of Du Bois, and HbA1c was converted from % to mmol/mol by the formula: HbA1c (mmol/mol) = (HbA1c%–2.15) × 10.929. Data are shown as mean values unless otherwise noted. ΔRER values are shown in a Forest plot (95% CI) in Figure 3.

Abbreviations: N/A, not available; NS, not significant; RCT, randomised controlled trial.

^a^ Three studies had two OW groups [29, 35, 44] and one study had two T2D groups [43] included in the same study, and for these, the data were combined according to the formulas provided in the Cochrane Handbook.

^b^ Converted/calculated.

^c^ From figure.

^d^ Median.

^e^ RER data only on *n* = 19.

^f^ RER data only on *n* = 17.

^g^ RER data only on *n* = 10.

The 35 studies using a ~ 40 mU/m^2^/min insulin infusion rate comprised 985 participants (256 Lean, 497 OW and 232 T2D participants) and included 16 cross‐sectional studies, 2 cohort studies, 11 randomised controlled trials (RCTs), and 6 clinical trials. The studies using insulin infusion of < 37 or > 43 mU/m^2^/min (*n* = 31) included 819 participants (238 Lean, 320 OW and 261 T2D) in 15 cross‐sectional studies, 8 RCTs, and 8 clinical trials. Only studies performing a euglycaemic clamp are presented in Tables 1 and S1, while results from isoglycaemic clamps (*n* = 2) are presented in Section 3.5.

### 3.2 Participant Characteristics

57% of the participants from the ~40 mU/m^2^/min studies that presented sex distribution (*n* = 32) were male (Lean 52%, OW 51%, T2D 76%). The median (range) of the presented age in the studies (*n* = 35) was 47 (16–66) years, with Lean (30 (16–54) years) being younger than OW (49 (24–66) years) and T2D (54 (44–64) years). The BMI (*n* = 34) was 22 (18–25) kg/m^2^ in Lean, 30 (26–41) kg/m^2^ in OW, and 30 (27–38) kg/m^2^ in T2D. HbA1c (*n* = 15) was 37 (36–37) mmol/mol in Lean, 36 (29–41) mmol/mol in OW, and 55 (50–89) mmol/mol in T2D. The studies using an insulin infusion rate of < 37 or > 43 mU/m^2^/min had similar characteristics, though a slightly lower percentage of male participants (50%) and a median age of 38 (13–64 years) (Table S1). 43% of the ~40 mU/m^2^/min studies presented HbA1c values; 86% of the studies including participants with T2D presented HbA1c values. This was similar in the other studies (40% of all studies and 85% of studies with T2D participants).

### 3.3 Basal RER

**FIGURE 2:**
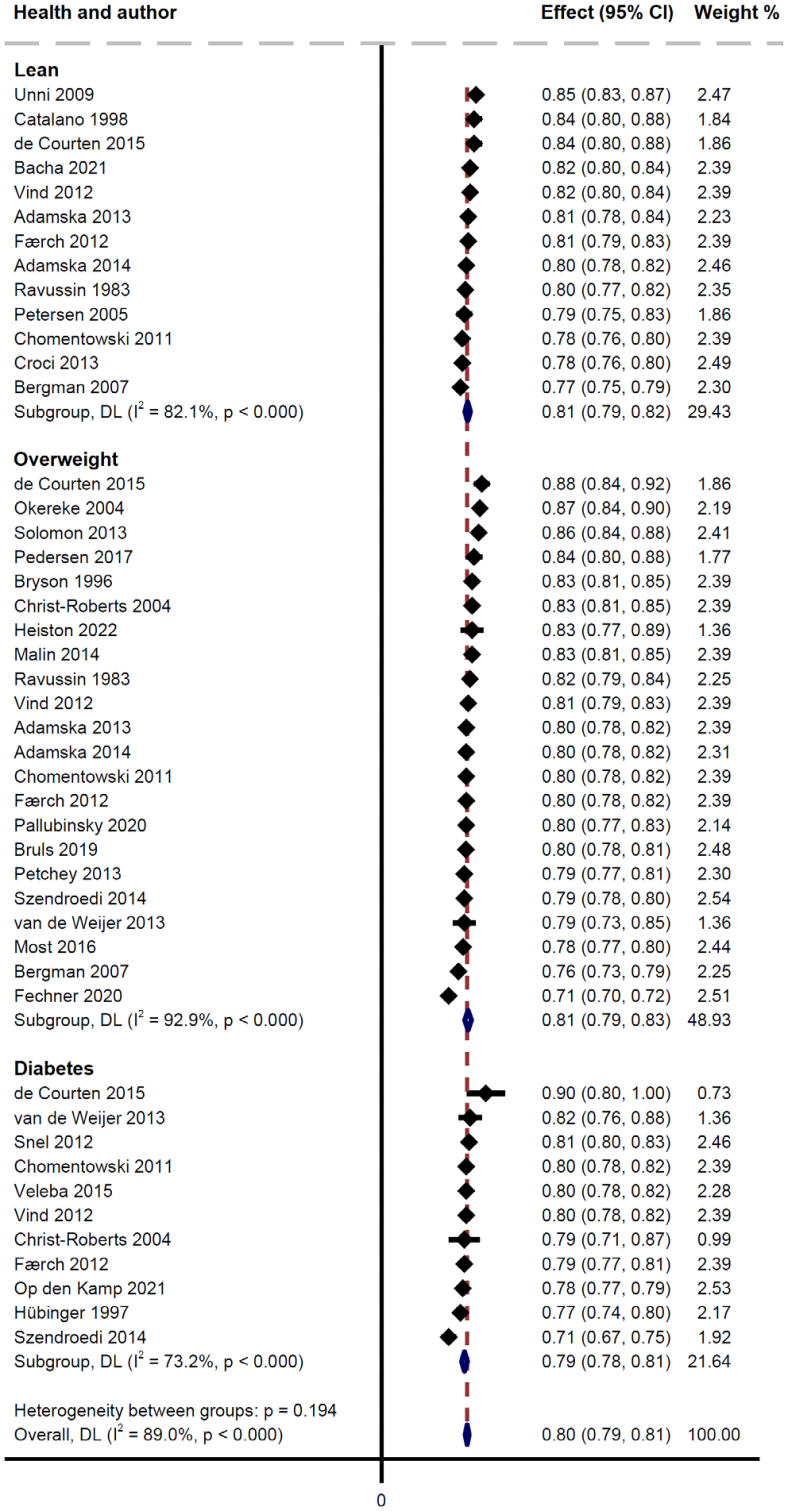
Studies included in the meta‐analysis displayed as a Forest plot of the basal RER value (Table 1). Indirect comparison of Lean, Overweight and Type 2 diabetes groups. Dotted red vertical line: Average RER of all studies. Heterogeneity between the three groups is substantial and no significant difference between them was found.

Basal RER in the studies with ~40 mU/m^2^/min infusion rate was not significantly different across the three population groups (*p* = 0.194). The overall statistical heterogeneity was high (*I* ^2^ = 89.0%). In Lean, the mean RER was 0.81 (95% CI: 0.79–0.82; *I* ^2^ = 82.1%; 13 groups), in OW it was 0.81 (95% CI: 0.79–0.83; *I* ^2^ = 92.9%; 22 groups), and in T2D it was 0.79 (95% CI: 0.78–0.81; *I* ^2^ = 73.2%; 11 groups) (Figure 2).

### 3.4 Insulin‐Stimulated Changes in RER

**FIGURE 3:**
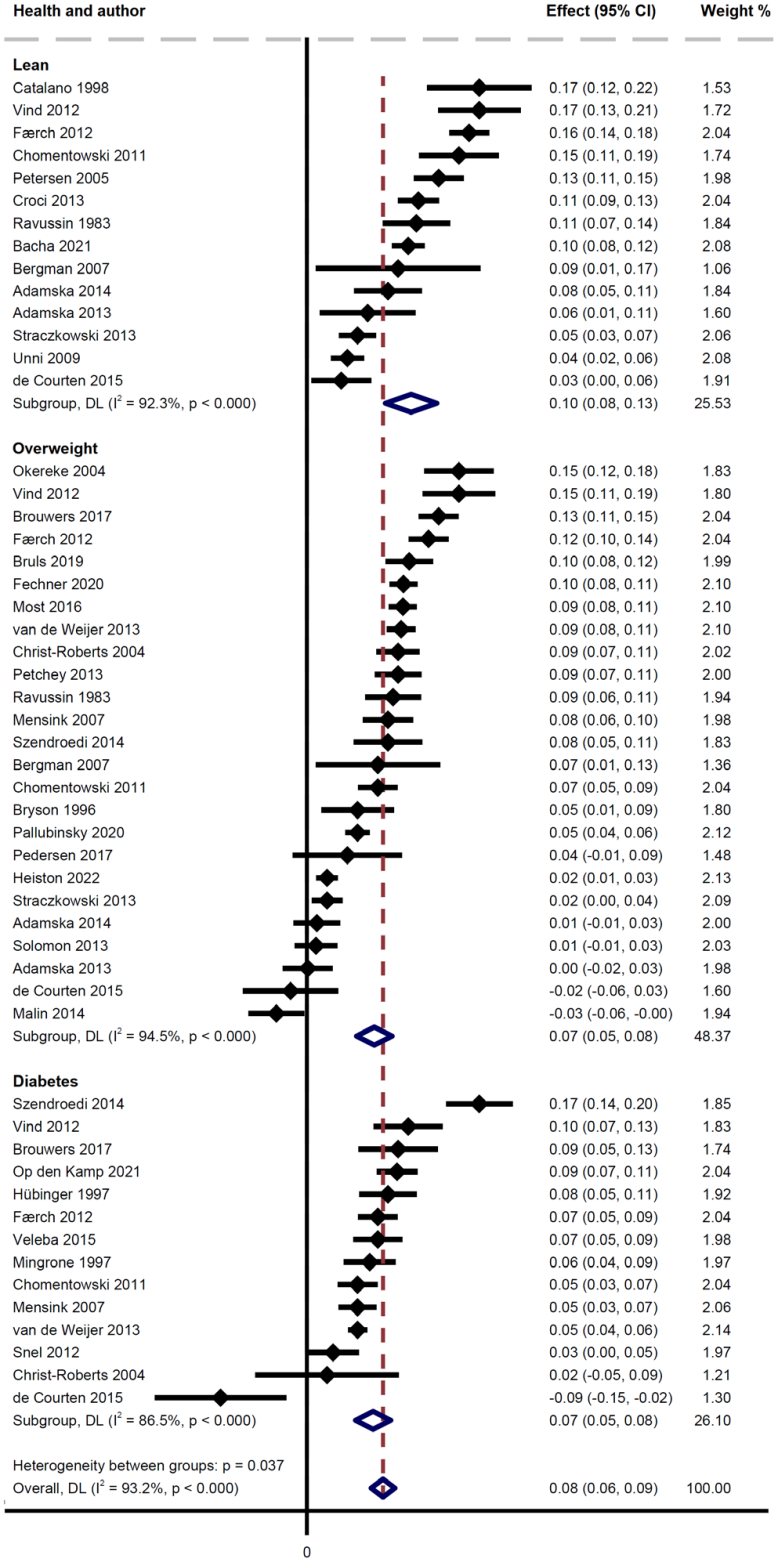
Studies included in the meta‐analysis displayed as a Forest plot of the ΔRER value (Table 1). Indirect comparison of Lean, Overweight and Type 2 diabetes groups. Zero: No change with insulin stimulation. Dotted red vertical line: Average ΔRER of all studies. A significant mean difference in ΔRER across the three population groups (*p* = 0.035) is found, but heterogeneity between the three groups is substantial.

The meta‐analysis based on ~40 mU/m^2^/min infusion rate showed a significant mean difference in ΔRER across the three population groups (*p* = 0.037) with a high level of statistical heterogeneity (*I* ^2^ = 93.2%). This difference was driven by a higher mean ΔRER in the Lean subgroup. In Lean, the mean ΔRER was 0.10 (95% CI: 0.08–0.13; *I* ^2^ = 92.3%; 14 groups), in OW it was 0.07 (95% CI: 0.05–0.08; *I* ^2^ = 94.5%; 25 groups), and in T2D it was 0.07 (95% CI: 0.05–0.08; *I* ^2^ = 86.5%; 14 groups) (Figure 3). The meta‐regression showed that only BMI was significantly associated with ΔRER, with a decrease of 0.0034 per BMI unit (*p* = 0.018), accounting for 11% of the total variance (see Appendix S1).

In total, 12 studies presented statistics comparing ΔRER between groups, five compared clamp RER between groups, and 16 studies reported statistics on within‐group changes from basal to insulin‐stimulated RER. Thirty‐two studies did not report statistical results relevant to this review.

### 3.5 Isoglycaemic Clamp

Two studies used an isoglycaemic clamp (i.e., clamping at the fasting plasma glucose level of the individual). Pelikánová et al. performed 2‐step isoglycaemic clamps with and without co‐Intralipid infusions on 10 male participants with T2D (46.4 years, BMI 26.4 kg/m^2^, HbA1c 52 mmol/mol). RER changed from 0.77 in the basal state to 0.81 (1 mU/kg/min) and 0.88 (10 mU/kg/min) with insulin stimulation [52].

Vind et al. compared the euglycaemic (Table 1) and isoglycaemic clamps in the T2D group of their study [49]. During isoglycaemic conditions, RER increased from 0.84 to 0.96 (i.e., ΔRER ~0.12), which erased the differences in clamp RER between T2D and both Lean and OW.

## 4 Discussion

### 4.1 The Importance of Basal RER

The overall impression of the basal RER from the 65 studies was that the groups in general did not differ (Tables 1 and S1). However, a large variability in the reported basal RER values was apparent, for example in lean subjects where a basal RER of 0.91 [53] was reported. Basal RER is typically measured after an overnight fast, and Miles‐Chan et al. found a positive relationship between the dietary proportion of carbohydrates and the post‐absorptive RER [54]. As such, diet composition may partly account for the variability in the studies, but 21 out of the 35 studies with ~40 mU/m^2^/min infusion rate studies did not provide information about the diet in the preceding day(s). Basal RER has been shown to correlate inversely with ΔRER [3] and therefore, it is important for the interpretation of a change in RER with insulin stimulation that the basal RER values are comparable across the studies. In the present meta‐analysis of basal RER, the mean values clustered closely around the overall mean value of 0.80 (Figure 2), albeit with a few exceptions, i.e. Fechner et al. [29] with a mean of 0.71 (95% CI: 0.70–0.72) in OW and de Courten et al. [27] and Szendroedi [46] with a mean of 0.90 (95% CI: 0.80–1.00) and 0.71 (95% CI: 0.67–0.75), respectively, in T2D.

### 4.2 Are Individuals With T2D Metabolically Inflexible?

Stull et al. found a positive correlation between metabolic flexibility and insulin sensitivity (*r* = 0.48, *p* < 0.0001) and observed that insulin sensitivity, fasting RER, plasma triglyceride concentrations, diabetes status, and ethnicity accounted for 71% of the variance in metabolic flexibility [55]. Skeletal muscle insulin sensitivity alone has been found to account for ~50% of the variance in metabolic flexibility [7].

In support of the effect of insulin sensitivity on metabolic flexibility, the Lean groups from the included studies using an insulin infusion rate of ~40 mU/m^2^/min cluster around a higher ΔRER than their OW and T2D counterparts, with T2D and OW being similar (Figure 3).

Mitochondrial respiratory capacity has been linked to insulin sensitivity and metabolic flexibility, where excess caloric intake leads to the accumulation of metabolic intermediates, inhibition of glucose and fatty acid uptake from the circulation, and early insulin resistance [56]. Excess lipid accumulation leading to lipotoxicity has been suggested to result in decreased mitochondrial vitality and impaired metabolic flexibility [56, 57]. However, in a convincing series of experiments, Holloszy et al. have shown that neither a low mitochondrial mass nor a high‐fat diet is mechanistically linked to insulin resistance [58, 59, 60, 61]. Furthermore, van de Weijer et al. found that in overweight individuals with and without T2D, the relationship between mitochondrial capacity and metabolic flexibility disappeared when correcting for whole‐body glucose disposal [50].

The meta‐analysis revealed significant differences in the mean ΔRER across Lean, OW, and T2D groups at an infusion rate of ~40 mU/m^2^/min, driven by a higher ΔRER in the Lean group. However, the meta‐analysis results were associated with high levels of statistical heterogeneity (Figure 3), suggesting substantial between‐study methodological heterogeneity, which may be due to variations in population characteristics and measurement techniques. Therefore, the meta‐analysis results should be interpreted with caution.

29% of the Lean studies presented ΔRER values lower than the mean of the T2D values. Choosing a threshold for metabolic flexibility in this regard seems unfeasible and the question remains whether we can aptly call one of the groups more metabolically flexible than the others. On the other hand, it is clear that the insulin‐resistant T2D group does not stand out as particularly less metabolically flexible than the OW group.

4.3

#### 4.3.1 Participant Characteristics: Health, Glycaemic Control, Age, Sex and BMI

**FIGURE 4:**
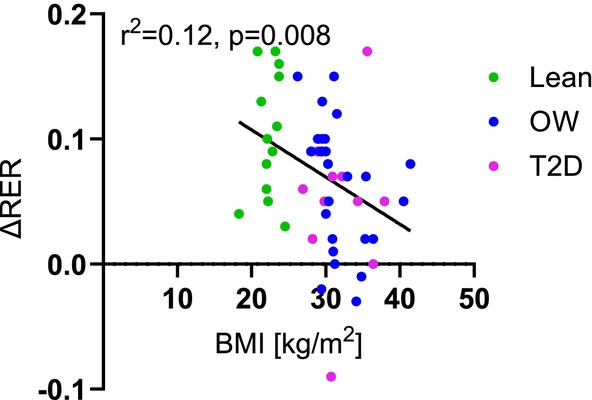
The correlation between ΔRER (insulin‐stimulated–basal) and body mass index (BMI). Green dots are Lean, blue dots are Overweight, and pink dots are Type 2 diabetes groups. Pearson's correlation coefficient is shown. A significant correlation was detected.

The large variability may be explained by differences in participants or methods used. The Lean group is, in all studies, considered healthy, although one study examined lean offspring of obese and lean parents [62]. The OW groups are more diverse, even though only healthy groups were included in the analysis. It has long been known that the distribution of adipose tissue makes a difference in metabolic health, but Nelleman et al. compared participants with upper vs. lower body overweight and found no differences between the groups in basal or clamp RER [63]. BMI is the one thing separating the Lean and OW groups, and in the present study, we included lean participants with a BMI < 25 kg/m^2^ and overweight participants with a BMI > 25 kg/m^2^. This means that the participants with the highest BMI in the Lean group are very similar to the participants with the lowest BMI in the OW group, possibly diluting the group effect. With pooled data from all three groups, we found a weak (*r* ^2^ = 0.12), albeit significant (*p* = 0.008) negative correlation between ΔRER and increasing BMI (Figure 4). Furthermore, our meta‐regression analysis, which included age, sex, and BMI as covariates, identified BMI as the only significant predictor. Specifically, each unit increase in BMI was associated with a 0.0034 decrease in ΔRER, explaining 11% of the total variance. However, the unexplained statistical heterogeneity remained high, indicating the need for caution when interpreting these results.

Individuals with T2D can differ in their glycaemic control, medical treatment, and co‐morbidities. Thus, eight studies [19, 37, 48, 49, 64, 65, 66, 67] presented mean or median HbA1c values fulfilling the International Diabetes Federation (IDF)'s recommended target below 53 mmol/mol [68], while three studies presented HbA1c values above 64 mmol/mol [26, 31, 69], which is characterised as “generally unacceptable” according to the IDF [68].

**FIGURE 5:**
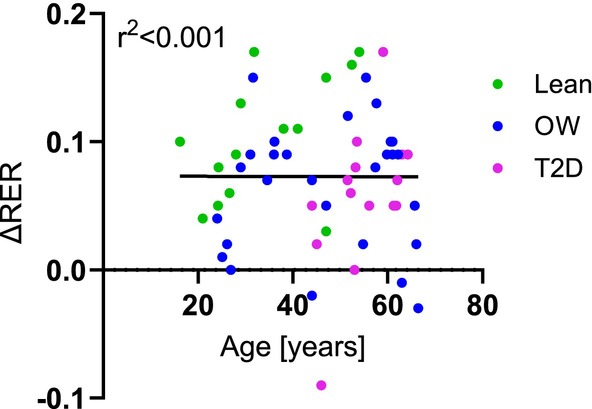
The correlation between ΔRER (insulin‐stimulated–basal) and age in the studies. Green dots are Lean, blue dots are Overweight, and pink dots are Type 2 diabetes groups. Pearson's correlation coefficient is shown. No significant correlation was detected.

Three studies in this review examined adolescents, and three studies presented mean or median ages of 65 years or higher. In general, the Lean studies reported younger age groups than OW and T2D, however, we found no correlation between age and ΔRER in the ~40 mU/m^2^/min studies (*r* ^2^ < 0.001, *p* = 0.99) (Figure 5).

Age, sex and BMI as covariates were analysed by meta‐regression (Appendix S1) which reinforced the conclusion that age and sex do not contribute to the variance in effect sizes with this dataset.

Most studies reported the distribution of men and women, and in the Lean groups, the distribution was equal between the two sexes, while two‐thirds of the T2D groups were men. Sparks et al. found that women were more metabolically flexible than men, with anti‐inflammatory adipose tissue macrophage activation and a greater capacity to suppress free fatty acids in response to insulin stimulation [70]. However, Stull et al. did not find sex to be a significant predictor of metabolic flexibility [55].

#### 4.3.2 Methodological Considerations: Diet, Environment and Equipment

In addition to the biological characteristics of the participants, methodological considerations play a role as well. Diet, energy balance, and fasting duration may affect basal RER [71], which is important for the magnitude of the RER increase during insulin stimulation. Likewise, a different period of medication discontinuation may affect results.

Here we decided to include only studies using whole‐body indirect calorimetry, which is often assessed with metabolic carts. Importantly, Galgani et al. touched upon problems of insufficient accuracy and reliability of metabolic carts [3]. Leg RQ has also been used to assess metabolic flexibility [2], and leg RQ has previously been found to increase significantly with insulin stimulation (and thereby glucose clearance rates), and similarly in obese patients with and without T2D [72]. Of note, the increase does not differ between trained and untrained legs in the two groups despite a massive difference in insulin sensitivity between the legs [72, 73].

#### 4.3.3 Methodological Considerations: Insulin Infusion Rate and Euglycaemic Versus Isoglycaemic Clamp

A high compared to a low insulin infusion rate will augment glucose disposal and is expected to result in a higher ΔRER and leg RQ, as seen in the studies using multiple insulin infusion rates [37, 72, 73, 74, 75]. However, the prevailing plasma glucose concentration during the clamp may also have an impact. A comparison of euglycaemic (plasma glucose levels of typically 5–5.5 mmol/L) with isoglycaemic (plasma glucose levels at the fasting level of each individual) clamps was not possible due to only two studies fulfilling our criteria. However, Vind et al. performed both euglycaemic and isoglycaemic clamps in participants with T2D, and when studied in the latter situation, all variables of insulin‐stimulated glucose metabolism were normalised compared with the weight‐matched control group [49]. These results beg the question of whether the isoglycaemic clamp should be considered when examining participants with T2D, as the euglycaemic clamp does not represent their habitual living conditions. In support, leg RQ (via arterial and femoral venous catheterisation) before and during isoglycaemic clamps increases similarly in T2D and matched controls with increasing insulin concentrations with no group difference [72, 73]. Furthermore, we have recently studied participants with T2D and an overweight control group during isoglycaemic conditions (ΔRER 0.02 in both groups during 40 mU/m^2^/min and 0.12 in the controls and 0.11 in T2D during 400 mU/m^2^/min, unpublished data). Galgani [3] has argued that metabolic flexibility exists at different levels (intake, circulation, tissue/cellular and mitochondrial) of the organism and that it is essential to establish which level is considered to understand which factors determine the interindividual variability. He found that when accounting for tissue glucose disposal, metabolic flexibility was similar between groups with different levels of insulin sensitivity. An advantage of the isoglycaemic clamp is that pre‐clamp insulin infusion in participants with T2D is avoided, equalising the insulin stimulus between the study groups, and the individual habitual metabolic state is preserved.

#### 4.3.4 Methodological Considerations: Methods for Estimating Metabolic Flexibility

Measurement of whole‐body gas exchange during a hyperinsulinemic, euglycaemic clamp is by far the most used method for assessing metabolic flexibility in patients with obesity and/or T2D and can be considered the gold standard.

The ability to shift fuel for oxidation can also be tested during a graded step‐wise exercise test with simultaneous gas exchange measurements. Although the method is being used more frequently now, there is a risk of selection bias because some exercise capacity is required of the participants. Furthermore, the number of studies that include patients with T2D and appropriate controls is limited.

Similarly, changes in RER values can be seen during a meal test, reflecting the oxidation of the ingested food. However, comparisons between studies would require identical meals (form, size and composition) and variation in gastric emptying between the subjects will be major confounders.

More advanced techniques like metabolomic and flux analysis using stable isotope tracers (glucose and/or fatty acids) would provide insight into substrate preferences during fasting, feeding, or insulin stimulation by calculation of substrate turnover and oxidation rates. While these techniques have been used in the study groups of interest in the present review, the number of individual studies is not sufficient to form a basis for a meta‐analysis.

### 4.4 Consensus on Data Presentation

We excluded 69% (*n* = 161) of the retrieved reports in our literature search because we could not obtain the ΔRER value. This was mostly in older studies but also the case for 47% (*n* = 7) of the studies published within the last 5 years. For transparency and the sake of comparison, the presentation of RER values (or the V̇O_2_ and V̇CO_2_) is essential and should be the standard.

We originally wanted to correlate insulin sensitivity with ΔRER but were unable due to different presentations of the data. Glucose infusion rate (GIR), glucose clearance, and the M‐value are used interchangeably as measures of insulin sensitivity, and they can be presented relative to body weight or fat‐free mass (FFM). While conversions are possible, they require information such as body mass, fat‐free mass, plasma glucose, and/or plasma insulin depending on the presentation format. However, these variables were not always shown.

Based on this we suggest that the minimum of participant characteristics presented in a clamp study entails the body weight, BMI, and fat‐free mass (if applied) of the participants. Insulin sensitivity should preferably be presented as either GIR (per bodyweight or FFM) or glucose clearance (per bodyweight or FFM). Additionally, both plasma glucose and insulin at basal and during the steady state of the clamp should be reported as a validation of the clamp procedure.

## 5 Conclusion

Individuals with T2D and non‐diabetic individuals with overweight displayed similar degrees of metabolic flexibility, while lean participants were more flexible. This indicates that metabolic flexibility is not linked to the T2D diagnosis as such, but overweight plays a role, which does, however, not necessarily imply a link to insulin sensitivity. Due to great variability in the study characteristics and results, assigning a threshold indicating a switch to metabolic *in*flexibility does not seem feasible. We propose that the isoglycaemic clamp should be used when examining individuals with T2D since this clamping method more closely resembles their (patho‐)physiological habitus, and we have suggested the minimum of data that should be presented in a clamp study with indirect calorimetry.

## Author Contributions

All authors contributed significantly to the work. Conception (F.D.); Selection (M.H., K.K.L.) and data extraction (M.H., K.K.L., M.B.S.); Metaanalysis and graphics (M.B.S.). Draft (M.H.) and final manuscript (M.H., K.K.L., M.B.S., F.D.).

## Disclosure

The authors have nothing to report.

## Conflicts of Interest

The authors declare no conflicts of interest.

## Supporting information


Appendix S1.

## Data Availability

The data that support the findings of this study are available on request from the corresponding author. The data are not publicly available due to privacy or ethical restrictions.
