# Supplementary material for: Are Individuals With Type 2 Diabetes Metabolically Inflexible? A Systematic Review and Meta‐Analysis
Source: Endocrinol Diabetes Metab. 2025 May 2;8(3):e70044. doi: 10.1002/edm2.70044 (PMC12048703; doi:10.1002/edm2.70044)
Supplement: Supplementary file 1 — Appendix S1. [file EDM2-8-e70044-s001.docx]

# Supplementary material

## Complete literature search strings for the three Pubmed searches

Lean: ((((("normal body weight"[Text Word]) OR ("body weight, normal"[Text Word])) OR ("normal weight"[Text Word])) OR ("ideal body weight"[Text Word])) OR (lean[Text Word])) OR ("Ideal Body Weight"[Mesh]) AND ((((( "Adaptation, Physiological/metabolism"[Mesh] OR "Adaptation, Physiological/physiology"[Mesh] )) OR "Basal Metabolism"[Mesh]) OR "Calorimetry, Indirect"[Mesh]) OR ((((((((((("basal metabolism"[Text Word]) OR ("resting metabolic rate*"[Text Word])) OR ("indirect calorimetr*"[Text Word])) OR ("respiratory gas exchange"[Text Word])) OR ("respiratory gas"[Text Word])) OR (RER[Text Word])) OR (RQ[Text Word])) OR ("resting respiratory exchange rat*"[Text Word])) OR ("metabolic flexibility"[Text Word])) OR ("metabolic inflexibility"[Text Word])) OR ("substrate utilization"[Text Word]))) AND (("Glucose Clamp Technique"[Mesh]) OR ((((((("Glucose Clamp Technique*"[Text Word]) OR ("euglycemic clamp*"[Text Word])) OR ("euglycaemic clamp*"[Text Word])) OR ("isoglycemic clamp*"[Text Word])) OR ("isoglycaemic clamp*"[Text Word])) OR ("hyperinsulinemic clamp*"[Text Word])) OR ("hyperinsulinaemic clamp*"[Text Word])))

Overweight: ((Overweight[MeSH Terms]) OR (Obesity[MeSH Terms])) OR ((Overweight[Text Word]) OR (Obes*[Text Word])) AND ((((( "Adaptation, Physiological/metabolism"[Mesh] OR "Adaptation, Physiological/physiology"[Mesh] )) OR "Basal Metabolism"[Mesh]) OR "Calorimetry, Indirect"[Mesh]) OR ((((((((((("basal metabolism"[Text Word]) OR ("resting metabolic rate*"[Text Word])) OR ("indirect calorimetr*"[Text Word])) OR ("respiratory gas exchange"[Text Word])) OR ("respiratory gas"[Text Word])) OR (RER[Text Word])) OR (RQ[Text Word])) OR ("resting respiratory exchange rat*"[Text Word])) OR ("metabolic flexibility"[Text Word])) OR ("metabolic inflexibility"[Text Word])) OR ("substrate utilization"[Text Word]))) AND (("Glucose Clamp Technique"[Mesh]) OR ((((((("Glucose Clamp Technique*"[Text Word]) OR ("euglycemic clamp*"[Text Word])) OR ("euglycaemic clamp*"[Text Word])) OR ("isoglycemic clamp*"[Text Word])) OR ("isoglycaemic clamp*"[Text Word])) OR ("hyperinsulinemic clamp*"[Text Word])) OR ("hyperinsulinaemic clamp*"[Text Word])))

Type 2 diabetes: (((((((((((("Type 2 diabetes"[Text Word]) OR ("Type 2 diabetes mellitus"[Text Word])) OR ("Diabetes type 2"[Text Word])) OR ("Diabetes mellitus type 2"[Text Word])) OR ("Non-insulin-dependent diabetes"[Text Word])) OR ("non-insulin-dependent diabetes mellitus"[Text Word])) OR ("noninsulin-dependent diabetes"[Text Word])) OR ("noninsulin-dependent diabetes mellitus"[Text Word])) OR (T2D[Text Word])) OR (T2DM[Text Word])) OR ("Diabetes Mellitus, Type 2"[Mesh])) AND ((((( "Adaptation, Physiological/metabolism"[Mesh] OR "Adaptation, Physiological/physiology"[Mesh] )) OR "Basal Metabolism"[Mesh]) OR "Calorimetry, Indirect"[Mesh]) OR ((((((((((("basal metabolism"[Text Word]) OR ("resting metabolic rate*"[Text Word])) OR ("indirect calorimetr*"[Text Word])) OR ("respiratory gas exchange"[Text Word])) OR ("respiratory gas"[Text Word])) OR (RER[Text Word])) OR (RQ[Text Word])) OR ("resting respiratory exchange rat*"[Text Word])) OR ("metabolic flexibility"[Text Word])) OR ("metabolic inflexibility"[Text Word])) OR ("substrate utilization"[Text Word])))) AND (("Glucose Clamp Technique"[Mesh]) OR ((((((("Glucose Clamp Technique*"[Text Word]) OR ("euglycemic clamp*"[Text Word])) OR ("euglycaemic clamp*"[Text Word])) OR ("isoglycemic clamp*"[Text Word])) OR ("isoglycaemic clamp*"[Text Word])) OR ("hyperinsulinemic clamp*"[Text Word])) OR ("hyperinsulinaemic clamp*"[Text Word])))

**Table S1** Characteristics of studies using insulin infusion rate <37 or >43 mU/m^2^/min during a hyperinsulinaemic euglycaemic clamp.

|  | | **Participant general characteristics** | | | | | | | **Clamp** | **Indirect calorimetry** | | |
| --- | --- | --- | --- | --- | --- | --- | --- | --- | --- | --- | --- | --- |
| **Author** | **Study type** | **Group** | **Number**  **n (m / f)** | **Age**  **Years** | **Weight**  **kg** | | **BMI**  **Kg/m^2^** | **HbA1c**  **mmol/mol** | **Insulin infusion rate mU/m^2^/min** | **Basal RER** | **ΔRER** | **Significance**  **P-value** |
| [1] | Clinical trial | **OW** | 10 (10 / 0) | 22.5 | 80.9 | 27.9 | | N/A | 80 | 0.72 | 0.07 | N/A |
| [2] | Cross-sectional | **OW** | 24 (8 / 16) | 15.2 | 81.3 | 30.8 | | 36^c^ | 80 | 0.81 | 0.08 | <0.05  ΔRER T2D vs OW |
|  |  | **T2D** | 28 (12 / 16) | 15.6 | 96.3 | 35.6 | | 44^c^ | 80 | 0.79 | 0.07 |  |
| [3] | Cross-sectional | **OW** | 14 (14 / 0) | 56^m^ | N/A | 30^m^ | | 35^m^ | 1.5 mU/kg/min | 0.78^m^ | 0.15^f^ | <0.01  ΔRER T2D vs OW |
|  |  | **T2D** | 14 (14 / 0) | 52^m^ | N/A | 32^m^ | | 53^m^ | 1.5 mU/kg/min | 0.79^m^ | 0.09^f^ |  |
| [4] | Cross-sectional | **Lean** | 17 (0 / 17) | 22.8 | 63.8 | 23.7 | | N/A | 80 | 0.84 | 0.14^c^ | <0.05  Clamp RER T2D vs Lean and OW |
|  |  | **OW** | 12 (0 / 12) | 46.1 | 86.5 | 33.3 | | N/A | 80 | 0.82 | 0.13^c^ |  |
|  |  | **T2D** | 27 (0 / 27) | 58.2 | 100.0 | 33.9 | | N/A | 80 | 0.83 | 0.06 |  |
| [5] | RCT | **OW** | 20 (20 / 0) | 60 | 104.6 | 33.3 | | 40 | 24^c^ | 0.80 | 0.03 | <0.01  Basal vs clamp RER |
|  |  | **OW** | 20 (20 / 0) | 58 | 104.8 | 32.4 | | 38 | 24^c^ | 0.80 | 0.03 | <0.01  Basal vs clamp RER |
| [6] | Clinical trial | **OW** | 42 (17 / 25) | 54.9 | 94.4 | 32.7 | | N/A | 80 | 0.83 | 0.10 | <0.0001  ΔRER T2D vs OW |
|  |  | **T2D** | 59 (26 / 33) | 60.0 | 95.9 | 33.8 | | N/A | 80 | 0.83 | 0.06 |  |
| [7] | RCT | **T2D** | 12 (9 / 3) | 60 | 93.8 | 31.2 | | 45 | 27^c^ | 0.81 | 0.01^c^ | N/A |
|  |  | **T2D** | 12 (6 / 6) | 64 | 88.6 | 30.3 | | 51 | 26^c^ | 0.81 | 0.05^c^ |  |
| [8] | Cross-sectional | **Lean** | 16 (N/A) | N/A | 73.8 | 24.3 | | N/A | 120 | 0.82 | 0.14 | 0.07  T2D vs lean |
|  |  | **T2D** | 9 (N/A) | N/A | 79.0 | 25.4 | | 81^c^ | 120 | 0.82 | 0.11 |  |
| [9] | Cross-sectional | **Lean** | 14 (6 / 8) | 26 | N/A | 20.7 | | 32^c^ | 120 | 0.91 | -0.01 | N/A |
| [10] | Clinical trial | **Lean** | 9 (9 / 0) | 24^m^ | N/A | 21.5 | | 30.4 | 30 | 0.82 | 0.04^c^ | N/A |
|  |  | **OW** | 9 (9 / 0) | 24^m^ | N/A | 35.7 | | 33.7 | 30 | 0.80 | 0.01^c^ |  |
| [11] | RCT | **OW** | 10 (0 / 10) | 30.4 | N/A | 37.0 | | N/A | 0.4 mU/kg/min | 0.82^f^ | 0.06^c^ | N/A |
| [12] | RCT | **T2D** | 19 (11 / 8) | 53 | N/A | 32.4 | | 43 | 1.5 mU/kg/min | 0.85 | 0.06 | <0.01  Basal vs clamp RER |
| [13] | Cross-sectional | **Lean** | 24 (0 / 24) | 24.1 | 57.3 | 21.0 | | N/A | 28^c^ | 0.81^f^ | 0.06 | N/A |
| [14] | Cross-sectional | **OW** | 17 (17 / 0) | 51 | 88 | 28.8 | | N/A | 80 | 0.85 | 0.12^c^ | N/A |
| [15] | Cross-sectional | **Lean** | 21 (11 / 10) | 23 | N/A | 20.9 | | N/A | 6 pmol/kg/min | 0.79 | 0.08 | <0.05  Basal vs clamp RER |
|  |  | **Lean** | 23 (7 / 16) | 22 | N/A | 22.0 | | N/A | 6 pmol/kg/min | 0.78 | 0.09 | <0.05  Basal vs clamp RER |
| [16] | Cross-sectional | **OW** | 12 (7 / 5) | 14.6 | N/A | 34.6 | | N/A | 80 | 0.78 | 0.10 | <0.001  Basal vs clamp RER |
| [17] | RCT | **Lean** | 8 (8 / 0) | 23 | N/A | 23.8 | | N/A | 1.4 mU/kg/min | 0.78^f^ | 0.12^f^ | <0.001  Basal vs clamp RER |
|  |  | **Lean** | 9 (9 / 0) | 23 | N/A | 23.9 | | N/A | 1.4 mU/kg/min | 0.82^f^ | 0.07^f^ | <0.001  Basal vs clamp RER |
| [18] | RCT | **OW** | 8 (0 / 8) | 53 | 91.3 | 35.7 | | N/A | 20 | 0.81 | 0.04 | N/A |
|  |  |  |  |  |  |  |  |  | 120 | 0.81 | 0.11 | N/A |
| [19] | Cross-sectional | **Lean** | 39 (20 / 19) | 13 | 47.8 | 19 | | 34^c^ | 80 | 0.85 | 0.08^c^ | 0.005  Clamp RER Lean vs OW  <0.001  Clamp RER T2D vs Lean and OW |
|  |  | **OW** | 64 (34 / 30) | 13.4 | 92.5 | 34.6 | | 34^c^ | 80 | 0.84 | 0.08^c^ |  |
|  |  | **T2D** | 17 (8 / 9) | 15.3 | 107.0 | 37.2 | | 51^c^ | 80 | 0.82 | 0.05^c^ |  |
| [20] | Cross-sectional | **Lean** | 10 (0 / 10) | 39 | N/A | 22.9 | | N/A | 0.6 mU/kg/min | 0.79 | 0.13^c^ | <0.001  Basal vs clamp RER within all groups  NS  Basal and clamp RER between all groups |
|  |  | **OW** | 10 (0 / 10) | 42 | N/A | 32.3 | | N/A | 0.6 mU/kg/min | 0.78 | 0.12^c^ |  |
|  |  | **OW** | 11 (0 / 11) | 38 | N/A | 30.5 | | N/A | 0.6 mU/kg/min | 0.76 | 0.14^c^ |  |
| [21] | RCT | **T2D** | 24 (19 / 5) | 64.2 | N/A | 28.1 | | 51.7 | 10 | 0.78 | 0.03^c^ | N/A |
| [22] | Clinical trial | **Lean** | 13 (4 / 9) | 33 | 61 | 22.4 | | N/A | 47^c^ | 0.79 | 0.06 | <0.05  Basal vs clamp RER |
|  |  | **OW** | 13 (4 / 9) | 38 | 155 | 56.3 | | N/A | 47^c^ | 0.74 | 0.01 | NS  Basal vs clamp RER |
| [23] | Clinical trial | **OW** | 8 (5 / 3) | N/A | 96.2 | 31.4 | | N/A | 190^c^ | 0.80 | 0.06 | N/A |
|  |  | **T2D** | 7 (4 / 3) | N/A | 96.0 | 31.2 | | N/A | 190^c^ | 0.80 | 0.06 |  |
| [24] | Cross-sectional | **OW** | 10 (N/A) | 63 | 86 | 29 | | N/A | 93^c^ | N/A | 0.12 | <0.01  Basal vs clamp RER |
| [25] | Cross-sectional | **T2D** | 15 (4 / 11) | 45.1 | N/A | 35.6 | | 58^c^ | 1 mU/kg/min | 0.81^f^ | 0.07^f,c^ | N/A |
| [26] | Clinical trial | **T2D** | 8 (7 / 1) | 54 | 85.4^c^ | 28.7 | | 57^c^ | 429^c^ | 0.79 | 0.10^c^ | N/A |
| [27] | Clinical trial | **Lean** | 10 (7 / 3) | 45 | 75.6 | 24.3 | | 36^c^ | 80 | 0.81 | 0.09^c^ | N/A |
|  |  | **Lean** | 5 (2 / 3) | 49 | 72.6 | 24.5 | | 36^c^ | 80 | 0.81 | 0.10^c^ |  |
| [28] | Cross-sectional | **Lean** | 9 (0 / 9) | 35.6 | 63.4 | 21.9 | | N/A | 100 | 0.82 | 0.14^c^ | <0.0002  Basal vs clamp RER |
| [29] | RCT | **T2D** | 10 (10 / 0) | 52.4 | N/A | 30.1 | | 45.9 | 1 mU/kg/min | 0.82 | 0.05^c^ | N/A |
| [30] | Clinical trial | **OW** | 6 (2 / 4) | 34.8 | 110.0 | 37.0 | | N/A | 100 | 0.78 | 0.08 | N/A |
| [31] | Cross-sectional | **Lean** | 11 (11 / 0) | 28.7 | N/A | 24.1 | | 29^c^ | 1 mU/kg/min | 0.75 | 0.11 | N/A |
|  |  |  |  |  |  |  |  |  | 10 mU/kg/min | 0.75 | 0.16 |  |

Legend to Table S1: Thirty-one studies using insulin infusion rates below 37 or above 43 mU/m^2^/min during the clamp in lean and overweight (OW) individuals and patients with type 2 diabetes (T2D). Data are shown as mean values unless otherwise noted. Where possible, we converted insulin infusion rates to mU/m^2^/min (1 μU/mL=6.00 pmol/L) [32]. Body area was calculated by the formula of Du Bois, and HbA1c was converted from % to mmol/mol by the formula: HbA1c (mmol/mol) = (HbA1c% – 2.15) x 10.929. RCT=Randomized Controlled Trial; ^m^median; ^f^from figure; ^c^converted; N/A=Not Available; NS=Not Significant.

**Age, sex and BMI as covariates analysed by meta-regression**.

The impact of these variables was examined through meta-regression. The initial model included age, sex, and BMI as covariates, explaining 15.94% of the variance, based on 47 observations (Table S2). Age and BMI were found to be significant predictors, while sex was not significant.

In a stepwise approach, we removed sex from the model due to its lack of significance, which increased the number of observations to 51 and resulted in age also no longer being significant (Table S3). Consequently, age was excluded from the final model, leaving BMI as the only significant covariate (Table S4).

Furthermore, we conducted an additional exploratory meta-regression model including **age** and **sex** as the only covariates to further explore whether any collinearity or hidden interactions between these variables might influence the final results Table S5).

This analysis revealed that **neither age nor sex were significant predictors** of the effect size. Furthermore, the adjusted R² for this model was **0%**, indicating that these variables did not explain any of the between-study heterogeneity. This finding reinforces the conclusion that **age and sex do not contribute to the variance in effect sizes** within this dataset.

Table S2:


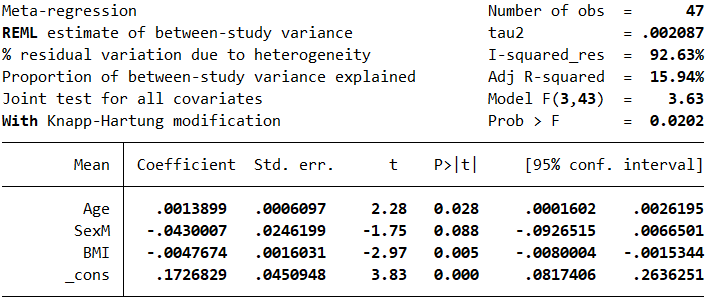


Table S3:


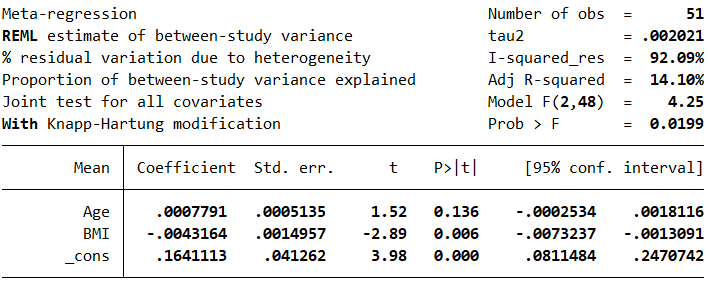


Table S4:


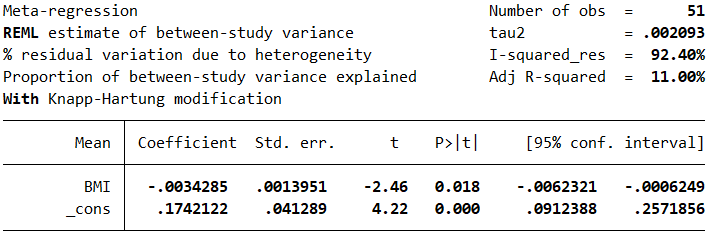


The final model revealed that BMI was associated with a decrease in ΔRER of 0.0034 per BMI unit, explaining 11% of the variance, based on 51 observations.

Table S5:


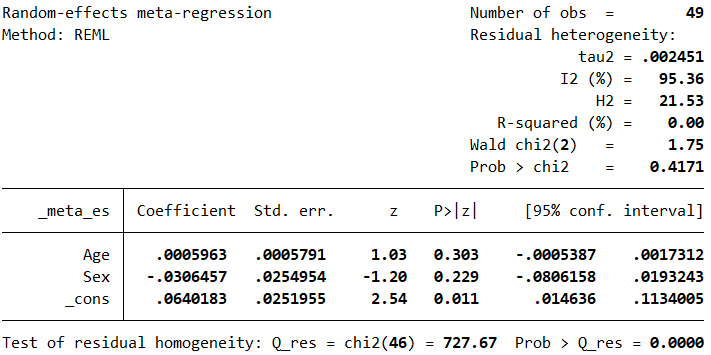


PRISMA checklist

| **Section/topic** | **#** | **Checklist item** | **Reported on page #** |
| --- | --- | --- | --- |
| **TITLE** | | |  |
| Title | 1 | Identify the report as a systematic review, meta-analysis, or both. | 1 |
| **ABSTRACT** | | |  |
| Structured summary | 2 | Provide a structured summary including, as applicable: background; objectives; data sources; study eligibility criteria, participants, and interventions; study appraisal and synthesis methods; results; limitations; conclusions and implications of key findings; systematic review registration number. | 3 |
| **INTRODUCTION** | | |  |
| Rationale | 3 | Describe the rationale for the review in the context of what is already known. | 5-6 |
| Objectives | 4 | Provide an explicit statement of questions being addressed with reference to participants, interventions, comparisons, outcomes, and study design (PICOS). | 5 |
| **METHODS** | | |  |
| Protocol and registration | 5 | Indicate if a review protocol exists, if and where it can be accessed (e.g., Web address), and, if available, provide registration information including registration number. | Not done |
| Eligibility criteria | 6 | Specify study characteristics (e.g., PICOS, length of follow-up) and report characteristics (e.g., years considered, language, publication status) used as criteria for eligibility, giving rationale. | 5-6 |
| Information sources | 7 | Describe all information sources (e.g., databases with dates of coverage, contact with study authors to identify additional studies) in the search and date last searched. | 5 |
| Search | 8 | Present full electronic search strategy for at least one database, including any limits used, such that it could be repeated. | Supplementary material |
| Study selection | 9 | State the process for selecting studies (i.e., screening, eligibility, included in systematic review, and, if applicable, included in the meta-analysis). | 5-6 |
| Data collection process | 10 | Describe method of data extraction from reports (e.g., piloted forms, independently, in duplicate) and any processes for obtaining and confirming data from investigators. | 5-6 |
| Data items | 11 | List and define all variables for which data were sought (e.g., PICOS, funding sources) and any assumptions and simplifications made. | 5-6 |
| Risk of bias in individual studies | 12 | Describe methods used for assessing risk of bias of individual studies (including specification of whether this was done at the study or outcome level), and how this information is to be used in any data synthesis. | Not applicable for baseline data |
| Summary measures | 13 | State the principal summary measures (e.g., risk ratio, difference in means). | 6 |
| Synthesis of results | 14 | Describe the methods of handling data and combining results of studies, if done, including measures of consistency (e.g., I^2^) for each meta-analysis. | 6 |
| Risk of bias across studies | 15 | Specify any assessment of risk of bias that may affect the cumulative evidence (e.g., publication bias, selective reporting within studies). | Not applicable for baseline data |
| Additional analyses | 16 | Describe methods of additional analyses (e.g., sensitivity or subgroup analyses, meta-regression), if done, indicating which were pre-specified. | Not applicable – no a priori protocol |
| **RESULTS** | | |  |
| Study selection | 17 | Give numbers of studies screened, assessed for eligibility, and included in the review, with reasons for exclusions at each stage, ideally with a flow diagram. | Figure 1 |
| Study characteristics | 18 | For each study, present characteristics for which data were extracted (e.g., study size, PICOS, follow-up period) and provide the citations. | 7 |
| Risk of bias within studies | 19 | Present data on risk of bias of each study and, if available, any outcome level assessment (see item 12). | Not applicable for baseline data |
| Results of individual studies | 20 | For all outcomes considered (benefits or harms), present, for each study: (a) simple summary data for each intervention group (b) effect estimates and confidence intervals, ideally with a forest plot. | Table 1 and figure 3 |
| Synthesis of results | 21 | Present results of each meta-analysis done, including confidence intervals and measures of consistency. | Figure 3 |
| Risk of bias across studies | 22 | Present results of any assessment of risk of bias across studies (see Item 15). | Not applicable for baseline data |
| Additional analysis | 23 | Give results of additional analyses, if done (e.g., sensitivity or subgroup analyses, meta-regression [see Item 16]). | Not done |
| **DISCUSSION** | | |  |
| Summary of evidence | 24 | Summarize the main findings including the strength of evidence for each main outcome; consider their relevance to key groups (e.g., healthcare providers, users, and policy makers). | 10-11 |
| Limitations | 25 | Discuss limitations at study and outcome level (e.g., risk of bias), and at review-level (e.g., incomplete retrieval of identified research, reporting bias). | 11-14 |
| Conclusions | 26 | Provide a general interpretation of the results in the context of other evidence, and implications for future research. | 14 |
| **FUNDING** | | |  |
| Funding | 27 | Describe sources of funding for the systematic review and other support (e.g., supply of data); role of funders for the systematic review. | 14 |

*From:*  Moher D, Liberati A, Tetzlaff J, Altman DG, The PRISMA Group (2009). Preferred Reporting Items for Systematic Reviews and Meta-Analyses: The PRISMA Statement. PLoS Med 6(7): e1000097. doi:10.1371/journal.pmed1000097

# References

[1] Amador M, Meza CA, McAinch AJ, King GA, Covington JD, Bajpeyi S. Exercise-Induced Improvements in Insulin Sensitivity Are Not Attenuated by a Family History of Type 2 Diabetes. Front Endocrinol (Lausanne). 2020;11:120.

[2] Bacha F, Bartz SK, Puyau M, Adolph A, Sharma S. Metabolic flexibility across the spectrum of glycemic regulation in youth. JCI Insight. 2021;6.

[3] Bodis K, Jelenik T, Lundbom J, Markgraf DF, Strom A, Zaharia OP, et al. Expansion and Impaired Mitochondrial Efficiency of Deep Subcutaneous Adipose Tissue in Recent-Onset Type 2 Diabetes. J Clin Endocrinol Metab. 2020;105:e1331-43.

[4] Broskey NT, Tam CS, Sutton EF, Altazan AD, Burton JH, Ravussin E, et al. Metabolic inflexibility in women with PCOS is similar to women with type 2 diabetes. Nutr Metab (Lond). 2018;15:75.

[5] Dollerup OL, Christensen B, Svart M, Schmidt MS, Sulek K, Ringgaard S, et al. A randomized placebo-controlled clinical trial of nicotinamide riboside in obese men: safety, insulin-sensitivity, and lipid-mobilizing effects. Am J Clin Nutr. 2018;108:343-53.

[6] Galgani JE, Heilbronn LK, Azuma K, Kelley DE, Albu JB, Pi-Sunyer X, et al. Metabolic flexibility in response to glucose is not impaired in people with type 2 diabetes after controlling for glucose disposal rate. Diabetes. 2008;57:841-5.

[7] Gormsen LC, Sondergaard E, Christensen NL, Brosen K, Jessen N, Nielsen S. Metformin increases endogenous glucose production in non-diabetic individuals and individuals with recent-onset type 2 diabetes. Diabetologia. 2019;62:1251-6.

[8] Gumbiner B, Thorburn AW, Henry RR. Reduced glucose-induced thermogenesis is present in noninsulin-dependent diabetes mellitus without obesity. J Clin Endocrinol Metab. 1991;72:801-7.

[9] Hardin DS, LeBlanc A, Para L, Seilheimer DK. Hepatic insulin resistance and defects in substrate utilization in cystic fibrosis. Diabetes. 1999;48:1082-7.

[10] Hogild ML, Bak AM, Pedersen SB, Rungby J, Frystyk J, Moller N, et al. Growth hormone signaling and action in obese versus lean human subjects. Am J Physiol Endocrinol Metab. 2019;316:E333-E44.

[11] Jorgensen JO, Pedersen SB, Borglum J, Moller N, Schmitz O, Christiansen JS, et al. Fuel metabolism, energy expenditure, and thyroid function in growth hormone-treated obese women: a double-blind placebo-controlled study. Metabolism. 1994;43:872-7.

[12] Kahl S, Nowotny B, Piepel S, Nowotny PJ, Strassburger K, Herder C, et al. Estimates of insulin sensitivity from the intravenous-glucose-modified-clamp test depend on suppression of lipolysis in type 2 diabetes: a randomised controlled trial. Diabetologia. 2014;57:2094-102.

[13] Karczewska-Kupczewska M, Straczkowski M, Adamska A, Nikolajuk A, Otziomek E, Gorska M, et al. Insulin sensitivity, metabolic flexibility, and serum adiponectin concentration in women with anorexia nervosa. Metabolism. 2010;59:473-7.

[14] Karhapaa P, Voutilainen E, Malkki M, Laakso M. Obese men with type IIB hyperlipidemia are insulin resistant. Arterioscler Thromb. 1993;13:1469-75.

[15] Lazarin MA, Bennini JR, Pereira CL, Astiarraga BD, Ferrannini E, Muscelli E. Normal insulin sensitivity in lean offspring of obese parents. Obes Res. 2004;12:621-6.

[16] Lee S, Rivera-Vega M, Alsayed HM, Boesch C, Libman I. Metabolic inflexibility and insulin resistance in obese adolescents with non-alcoholic fatty liver disease. Pediatr Diabetes. 2015;16:211-8.

[17] Lundsgaard AM, Fritzen AM, Sjoberg KA, Kleinert M, Richter EA, Kiens B. Small Amounts of Dietary Medium-Chain Fatty Acids Protect Against Insulin Resistance During Caloric Excess in Humans. Diabetes. 2021;70:91-8.

[18] Marlatt KL, Lovre D, Beyl RA, Tate CR, Hayes EK, Burant CF, et al. Effect of conjugated estrogens and bazedoxifene on glucose, energy and lipid metabolism in obese postmenopausal women. Eur J Endocrinol. 2020;183:439-52.

[19] Mihalik SJ, Michaliszyn SF, de las Heras J, Bacha F, Lee S, Chace DH, et al. Metabolomic profiling of fatty acid and amino acid metabolism in youth with obesity and type 2 diabetes: evidence for enhanced mitochondrial oxidation. Diabetes Care. 2012;35:605-11.

[20] Nellemann B, Gormsen LC, Sorensen LP, Christiansen JS, Nielsen S. Impaired insulin-mediated antilipolysis and lactate release in adipose tissue of upper-body obese women. Obesity (Silver Spring). 2012;20:57-64.

[21] Op den Kamp YJM, de Ligt M, Dautzenberg B, Kornips E, Esterline R, Hesselink MKC, et al. Effects of the SGLT2 Inhibitor Dapagliflozin on Energy Metabolism in Patients With Type 2 Diabetes: A Randomized, Double-Blind Crossover Trial. Diabetes Care. 2021;44:1334-43.

[22] Pereira JA, Lazarin MA, Pareja JC, de SA, Muscelli E. Insulin resistance in nondiabetic morbidly obese patients: effect of bariatric surgery. Obes Res. 2003;11:1495-501.

[23] Poynten AM, Markovic TP, Maclean EL, Furler SM, Freund J, Chisholm DJ, et al. Fat oxidation, body composition and insulin sensitivity in diabetic and normoglycaemic obese adults 5 years after weight loss. Int J Obes Relat Metab Disord. 2003;27:1212-8.

[24] Prior SJ, Ryan AS, Stevenson TG, Goldberg AP. Metabolic inflexibility during submaximal aerobic exercise is associated with glucose intolerance in obese older adults. Obesity (Silver Spring). 2014;22:451-7.

[25] Ramos-Roman MA, Pinero-Pilona A, Adams-Huet B, Raskin P. Comparison of type 1, type 2, and atypical ketosis-prone diabetes at 4 years of diabetes duration. J Diabetes Complications. 2006;20:137-44.

[26] Ratheiser K, Schneeweiss B, Waldhausl W, Fasching P, Korn A, Nowotny P, et al. Inhibition by etomoxir of carnitine palmitoyltransferase I reduces hepatic glucose production and plasma lipids in non-insulin-dependent diabetes mellitus. Metabolism. 1991;40:1185-90.

[27] Shannon C, Merovci A, Xiong J, Tripathy D, Lorenzo F, McClain D, et al. Effect of Chronic Hyperglycemia on Glucose Metabolism in Subjects With Normal Glucose Tolerance. Diabetes. 2018;67:2507-17.

[28] Toubro S, Western P, Bulow J, MacDonald I, Raben A, Christensen NJ, et al. Insulin sensitivity in post-obese women. Clin Sci (Lond). 1994;87:407-13.

[29] Vestergaard ET, Jessen N, Moller N, Jorgensen JOL. Unacylated Ghrelin Does Not Acutely Affect Substrate Metabolism or Insulin Sensitivity in Men With Type 2 Diabetes. J Clin Endocrinol Metab. 2019;104:2435-42.

[30] Webber J, Donaldson M, Allison SP, Fukagawa NK, Macdonald IA. The effects of weight loss in obese subjects on the thermogenic, metabolic and haemodynamic responses to the glucose clamp. Int J Obes Relat Metab Disord. 1994;18:725-30.

[31] Wohl P, Wohl P, Girman P, Pelikanova T. Inflexibility of energy substrate oxidation in type 1 diabetic patients. Metabolism. 2004;53:655-9.

[32] Knopp JL, Holder-Pearson L, Chase JG. Insulin Units and Conversion Factors: A Story of Truth, Boots, and Faster Half-Truths. J Diabetes Sci Technol. 2019;13:597-600.
